# Histone Deacetylase Inhibitors: Advancing Therapeutic Strategies in Hematological and Solid Malignancies

**DOI:** 10.3390/ph3082441

**Published:** 2010-08-04

**Authors:** Leigh Ellis, Roberto Pili

**Affiliations:** Roswell Park Cancer Institute, Genitourinary Program, Grace Cancer Drug Center, Buffalo, NY 14263, USA; E-Mail: Leigh.Ellis@roswellpark.org (L.E.)

**Keywords:** cancer, epigenetics, epigenetic therapy, HDAC, HDAC inhibitors, apoptosis, angiogenesis, autophagy, pre-clinical, clinical trial

## Abstract

Advancement in the understanding of cancer development in recent years has identified epigenetic abnormalities as a common factor in both tumorigenesis and refractory disease. One such event is the dysregulation of histone deacetylases (HDACs) in both hematological and solid tumors, and has consequently resulted in the development of HDAC inhibitors (HDACI) to overcome this. HDACI exhibit pleiotropic biological effects including inhibition of angiogenesis and the induction of autophagy and apoptosis. Although HDACI exhibit modest results as single agents in preclinical and clinical data, they often fall short, and therefore HDACI are most promising in combinational strategies with either standard treatments or with other experimental chemotherapies and targeted therapies. This review will discuss the induction of autophagy and apoptosis and the inhibition of angiogenesis by HDACI, and also pre-clinical and clinical combination strategies using these agents.

## 1. Introduction

The development and progression of cancer can evolve from the dysregulation of gene expression and function from a combination of genetic and epigenetic abnormalities [1]. One of the most common epigenetic abnormalities and a primary target for therapeutic intervention is the altered expression and/or cellular location of histone deacetylases (HDACs) in both hematological and solid malignancies [2]. 

Histone deacetylase inhibitors (HDACI) represent a new class of chemotherapy agents that target both histone and non-histone proteins. Two HDACI, vorinostat [3] and romidepsin [4], are now approved by the FDA for the treatment of cutaneous T cell lymphoma. HDACI mediate a wide range of biological effects including induction of apoptosis and autophagy and inhibition of angiogenesis [5,6]. Pre-clinical studies from both *in vitro* and *in vivo* models have demonstrated HDACI to be effective. Within the clinic HDACI as a monotherapy display modest anti-tumor activity with manageable side effects that are moderate and reversible. For this reason, it is predicted that the full potential of HDACI as anti-cancer therapies in the clinic will be achieved in combinational strategies with either standard treatments or with other experimental chemotherapies and targeted therapies. 

This review will summarize HDAC involvement in cancer and the role of HDACI in mediating induction of apoptosis and autophagy as well as the inhibition of angiogenesis. Further, the current literature on combinational strategies with HDACI to enhance these anti-tumor activities will be discussed. 

## 2. Histone Deacetylases and Cancer

HDACs are enzymes whose principle role is to oppose the activity of histone acetyl transferases (HATs) in regulating gene transcription and expression by removing acetyl groups from lysine residues of histone tails of chromatin and by the deacetylation of non-histone proteins [5,7]. The HDAC family is multiclass consisting of a total of 18 HDACs divided into four subgroups including: class I including HDAC 1, 2, 3, and 8 (localized to the nucleus); class II HDACs including 4, 5, 6, 7, 9 and 10 (localized to the both nucleus and cytoplasm); class III HDACs consists of sirtuins (1–7); and class IV includes HDAC 11, which displays features of both class I and II HDACs [8]. Class I, class II and class IV HDACs are structurally similar to the yeast proteins Hda1/Rpd3 and are zinc-dependent for their catalytic activity [9], while class III HDACs are Sir2 homologues and require NAD^+^ for their catalytic activity [10]. To influence gene transcription in both normal tissue and cancer, HDACs do not directly bind DNA but require interactions with other proteins in large multi-protein complexes. Due to the focus of this review only class I, II and IV HDACs will be elaborated on, as these classes of HDACs are currently being targeted in ongoing clinical trials (Table 1).

To date various studies have been conducted implicating aberrant expression of HDACs in tumorigenesis as well as progression to metastatic/refractory phenotypes. Examples of this include a recent study of class I HDAC expression patterns by Nakagawa *et al*. [11]. Included in this study were various cancer cell lines as well as a broad selection of primary human tissue samples representing lung, breast, ovary, esophageal, gastric, colon, thyroid, prostate and pancreatic cancers. Adjacent non-malignant tissue for each cancer sample was also included in this study. Overall, HDAC expression was found to be similar between non-malignant and malignant tissue samples, though certain tumor types including esophageal and prostate cancers displayed a trend in over-expressing class I HDACs [11]. More specifically, class I HDAC1, 2 and 3 are expressed within luminal cells of normal prostate tissue, while only weak expression is found in prostate basal cells [12]. In this study Weichert *et al.* also described a strong correlation with prostate adenocarcinoma and class I HDAC expression, showing strong positive nuclear staining for HDAC1, 2 and 3 in 70%, 74% and 95% of screened prostate adenocarcinomas [12]. Further, strong nuclear staining for HDAC1 protein expression has also been observed in castrate resistant cancers [13]. Halkidou *et al.* further demonstrated that castrate resistant prostate cancer expressed class II HDAC4 more predominately within the nucleus when compared to samples of benign prostate hyperplasia and androgen sensitive prostate cancers which maintained similar levels of HDAC4 expression within the cytosol, demonstrating a possible link between HDAC4 cellular location and loss of androgen sensitivity [14]. Of clinical relevance, Weichert *et al.* [12] also discuss the strong correlation between HDAC1 and HDAC2 expression with high grade Gleason score and increased proliferative potential. Further, using multivariate survival analysis, patients with increased HDAC2 expression also had decreased disease-free survival and concluded that HDAC2 was an independent prognostic factor. 

**Table 1:** Class I/II and IV HDACs; cellular localization and cancer association.

HDAC	Localization	Deregulation in cancer	Tumor
**Class I**			
HDAC1	Nucleus	Overexpression/underexpression	Esophageal, colon, prostate, CTCL
HDAC2	Nucleus	Overexpression/mutation	Prostate, colon, gastric, endometrial, CTCL
HDAC3	Nucleus	Overexpression	Prostate, colon
HDAC8	Nucleus	Overexpression	Colon
**Class IIa**			
HDAC4	Nucleus/Cytoplasm	Overexpression/underexpression/ mutation	Prostate, colon, breast
HDAC5	Nucleus/Cytoplasm	Underexpression	Colon, AML
HDAC7	Nucleus/Cytoplasm	Overexpression	Colon
HDAC9	Nucleus/Cytoplasm	Overexpression/underexpression	Medulloblastomas, astrocytomas
**Class IIb**			
HDAC6	Predominantly Cytoplasm	Overexpression	Breast, AML, CTCL
HDAC10	Predominantly Cytoplasm	Overexpression	Heptocellular Carcinoma
**Class IV**			
HDAC11	Nucleus/Cytoplasm	Overexpression	Breast

Altered expression and function of HDACs can arise from somatic mutations and germline polymorphisms. Studies including lung and breast cancer patients investigated the expression of germline variants of multiple HDACs and their correlation with disease risk. It was concluded that in lung cancer patients HDAC3, 4 and 5 and in breast cancer patients, HDAC2 and 5 were not associated with an increased risk of these respective cancers [15,16]. Further, recent studies have identified somatic mutations implicated in HDAC changes in expression and function. Ozdag *et al.* observed truncating mutations in HDAC2 in human epithelial cancers which were also associated with microsatellite instability [17]. This truncating mutation involving HDAC2 associated with microsatellite instability was also noted by Ropero *et al.* in multiple colonic, gastric and endometrial primary tumors. Functional assays conferred that this mutation was associated with resistance to HDAC inhibitor mediated apoptosis and inhibition of proliferation [18]. Another recent study by Sjoblom *et al.* involving a large scale sequencing analysis of breast and colorectal cancers indentified HDAC4 mutations occurred at a significant frequency only within breast cancer samples [19]. 

In hematological cancers recent studies have investigated the expression of HDACs in cutaneous T-cell lymphoma (CTCL). As stated earlier, CTCL represents the first malignancy in which the HDACI vorinostat (SAHA) [3] and romidepsin (Depsipeptide) [4] have been approved by the FDA for the treatment of this disease. In CTCL, a recent immunohistochemical study of HDAC1, 2 and 6 as well as histone H4 acetylation status was examined in 73 CTCL samples. Expression of HDAC1 was greatest, followed with HDAC2, and interestingly HADC6 and histone H4 acetylation were equally expressed. In aggressive forms however HDAC2 and histone H4 acetylation were more predominant than in indolent CTCL subtypes, while no differences were observed between HDAC1 and HDAC6. Increased HDAC6 expression did correlate to a favorable prognosis independent of subtype [20]. Other blood cancers also display aberrant recruitment of HDACs to specific loci through their interaction with proto-oncogenes with DNA binding ability to activate or repress gene transcription. An example of this involves a well characterized mechanism that occurs in acute promyelocytic leukemia (APL). Myeloid differentiation is a crucial biological process mediated by the retinoic acid receptor (RAR) which acts as a transcriptional regulator by heterodimerization with its binding partner RXR, allowing binding to retinoic acid response elements (RAREs) within the promoters of target genes [2]. In regards to APL, two chromosomal translocations can occur, t(15;17) and t(11;17), resulting in the production of two fusion proteins; RARα-PML and RARα-PLZF respectively. These fusion proteins retain high affinity to bind RAREs and HDACs and are non-responsive to retinoids, resulting in transcriptional silencing of RAR-target genes and the inhibition of cell differentiation [21,22,23,24]. Additional examples within hematological malignancies include the AML1-ETO fusion protein caused by the translocation t(8;21) in AML [25], and also in certain non-Hodgkin’s lymphomas where irregular expression of the oncogene Bcl-6 which recruits HDACs resulting in the transcriptional silencing of genes involved in cell cycle progression and apoptosis [8,25,26]. 

This is a brief overview of HDAC involvement in the development and progression of disease in cancer patients. For a more in depth reviews of this topic please refer to these latest papers and references within [2,27,28,29,30]. 

## 3. Histone Deacetylase Inhibitors

To date an extensive number of HDACI have been either purified from natural sources or synthetically developed. This has given rise to numerous HDACI being advanced to clinical development [2]. HDACI exist to target all classes of HDACs (Class I HDACs, class II HDACs, class IV HDACs and class III HDACs), though since the focus of this review is on class I, II and IV HDACs, so will be the discussion on specific HDACI towards these classes of HDACs. The majority of HDACI currently in clinical development targets multiple HDACs and because of this, affects many different molecular processes, including the inhibition of angiogenesis and the induction of autophagy and apoptosis [5,8]. 

HDACI were first thought to only mediate their anti-tumor mechanisms via changes in gene expression and studies have been published stating that ~5% of genes were regulated by HDACI at the transcriptional level [31,32,33,34,35,36]. These studies included diverse structural classes of HDACI, including sodium butyrate, vorinostat, MS-275, romidepsin and TSA. While these structural diverse HDACI mediate similar genes there was also an agent specific gene profile observed. Many of these genes were shown to be responsible for the biological effects of HDACI which was time and dose dependent [31,32,33,34,35,36]. Gene expression micro-array studies have been predominately conducted using cultured tumor cell lines and unfortunately to date limited information is available pertaining to gene expression profiles from patients treated with HDACI. However, two recent studies were published reporting transcriptional regulation activity by HDACI in cancer patients. A recent clinical trial consisting of lung cancer patients refractory to standard therapies were treated with romidepsin [37]. Of seventeen (17) patients available for gene analysis studies, it was reported that treatment of lung cancer patients with romidepsin resulted in increased acetylation of histone H4 and expression of the cyclin dependent kinase inhibitor p21, resulting in a best clinical response of stable disease (SD) [37]. Another clinical trial recently reported, consisted of patients with CTCL. Within this study patients were treated with panobinostat of which six (6) patients were available for further gene expression studies. Treatment with panobinostat resulted in increased acetylation of histone H3 and a set of commonly regulated genes in all patients. These genes represented biological processes such as apoptosis, angiogenesis and immune modulation. This clinical trial resulted in the best clinical outcome of a complete response (CR), with an overall 60% response rate [38]. Interestingly, both studies reported the majority of genes were transcriptionally repressed in response to their respective HDACI therapy, one of which was *CCND1*, which encodes for the oncogene Cyclin-D1. Unfortunately, because of limited patient number a concise correlation between gene expression patterns and clinical outcome could not be concluded, but does demonstrate what has already been noted from *in vitro* microarray studies. That is, structurally diverse HDACI can induce similar and independent transcriptional responses. 

As mentioned before, it is now realized that while HDACI have direct impact on gene transcription via their inhibition of HDAC function on histone tails, they also target gene transcription by indirect mechanisms via inhibiting HDAC interactions with non-histone proteins [39,40,41]. Examples of non-histone proteins that have been currently identified to have their function regulated by their acetylation status include the transcription factors p53 [42], NF-κB [41] and MYC [43] which have all been implemented to play important roles in tumorigenesis and anti-tumor responses. Further, proteins including Ku70 (that regulate DNA repair), Hsp90 (regulation of protein stability) and tubulin (cytoskeleton) which bare no direct role in gene expression can have their function determined by direct acetylation. A well documented example of non-histone protein regulation by acetylation is the chaperone protein Hsp90, which is targeted and deacetylated by HDAC6 [44]. Induction of acetylation by HDACI of Hsp90 results in the disassociation from its ‘client oncoproteins’ which in turn are targeted for degradation [44,45]. Accordingly, HDAC6 inhibition by HDACI is reported to result in the accumulation of polyubiquitinated proteins leading to cellular stress and apoptotic cell death [44]. 

## 4. HDACIs and Angiogenesis

Within tumors, new blood vessel formation (angiogenesis) can occur by sprouting from pre-existing vasculature which may be assisted by the recruitment of circulating cells such as bone marrow derived endothelial progenitor cells, macrophages and fibroblasts [46,47]. These cells along with malignant cells are able to secrete pro-angiogenic factors including vascular endothelial growth factor (VEGF) mediated by the hypoxia induced transcription factor-1 (HIF-1α), which induce tumor blood vessel formation [48].

Hypoxic conditions have been demonstrated to regulate HDAC function both directly and indirectly through mediating HDAC involvement in oxygen regulated gene expression and hypoxia-induced angiogenesis [49,50]. More specifically, it was demonstrated that under hypoxic conditions, various cell lines *in vitro* (both malignant and primary) exhibited increased expression of HDAC1, HDAC2 and HDAC3 mRNA and protein under hypoxic conditions [49]. In addition, the over-expression of HDAC1 mediated the reduced expression of p53 and pVHL, which resulted in the over-expression of HIF-1α and its transcriptional target VEGF, which was reversed by the use of the HDACI Trichostatin A (TSA) both *in vitro* and *in vivo* [49]. Later, Mahon *et al.* further described that the reduction in p53 and pVHL expression also resulted in reduction of factor inhibiting HIF-1α (FIH) allowing for the induction of angiogenesis in endothelial cells [50]. These initial reports demonstrated clearly that HDACs regulated HIF-1α activity indirectly under hypoxic conditions. Later, Fath *et al.* also showed that HIF-1α activity could be negatively regulated indirectly by the induction of p300 acetylation. This resulted in the suppression of HIF-1α transactivation activity and was also independent of p53 and pVHL [51]. Indirect regulation of HIF-1α could induce its degradation independent of pVHL and ubiquitin proteosomal degradation [52]. Moreover, the inhibition of HDAC6 by HDACI, demonstrated that hyperacetylation of Hsp90 resulted in the increased interaction and degradation of HIF-1α by Hsp70 [53].

HDACs also interact directly with HIF-1α to regulate its activity. It has been shown that HDAC1 and HDAC3 directly regulate HIF-1α stability and transcriptional activity via interaction with the ODDD of HIF-1α [54], though previous work conducted in our laboratory contradicts this report. Treatment of PC3 and C2 cell lines with the class I HDACI valproic acid [52] and MS275 (unpublished data) did not result in the loss of HIF-1α expression as shown by Kim *et al.* [54]. A possible explanation for these differences could be that HIF-1α direct interaction with HDAC1 and HDAC3 maybe cell specific. Furthermore, HDAC7, a class II HDAC, has strong interaction with factor inhibiting HIF-1α (FIH-1), but under hypoxic conditions HDAC7 translocates from the cytoplasm to the nucleus to bind HIF-1α and increase its transcriptional activity [55]. While HDAC7 has a role in regulating angiogenesis in tumor cells [55], it also influences angiogenesis in primary endothelial cells. Experiments where decreased expression of HDAC1 and HDAC7 in human umbilical vein endothelial cells (HUVECs) was induced, indicated that HDAC7 was necessary for the assembly of endothelial cell tube like structures *in vitro* [56]. Also, loss of HDAC7 expression also resulted in morphological changes and decreased endothelial cell migration, concurrent with increased expression platelet derived growth factor (PDGF)-B and its β receptor (PDGF-β) [56]. A further study by Qian *et al.* describes that HDAC4 and HDAC6 are vital for protein stability and transcriptional activity of HIF-1α. Exposure to the pan-HDACI LAQ824 induced HIF-1α acetylation. Inhibition of HDAC4 and HDAC6 demonstrated that these class II HDACs induced HIF-1α protein stability via proteosome-dependent pathway in renal cell carcinoma cell lines devoid of pVHL [52].

These studies demonstrate that HDACs play a critical role in hypoxic induced angiogenesis, and that targeting HDACs via their inhibition offers a new strategy in anti-cancer therapy through their ability to inhibit angiogenesis. Currently numerous pre-clinical and clinical studies exist which confirm a role for the anti-angiogenic activities of HDACI in the treatment of multiple tumors. The anti-angiogenic properties of HDACI have been associated with the alteration of numerous pro- and anti-angiogenic genes [57], which can be viewed in Table 2 and references there within. 

### 4.1. Combination Strategies with HDACI to Target Angiogenesis

**Figure 1:**
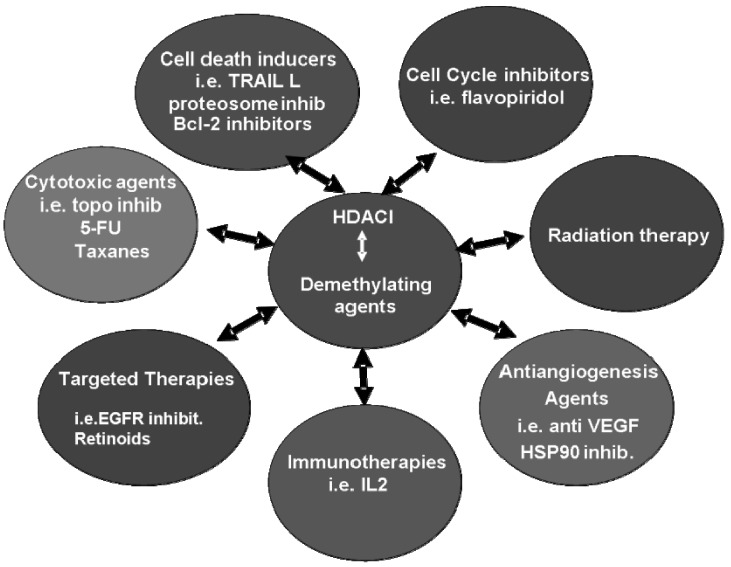
Schematic cartoon representing pre-clinical and clinical combination strategies with HDACI.

While HDACI are being combined with multiple anti-cancer agents (both novel and conventional) [5], only a few strategies targeting angiogenesis are currently under development that may exhibit promise to clinical translation (Figure 1). Results by Qian *et al.* described that the TKI PTK787/ZK222584 only exhibited anti-angiogenesis effect on endothelial cells while the HDACI LAQ824 targeted both endothelial cells and tumor epithelial cells [59]. Combination treatment of both agents resulted in better efficacy of inhibiting *in vitro* and *in vivo* VEGF-mediated angiogenesis. Furthermore, LAQ824 inhibited the expression of angiogenic genes including *angiopoietin-2*, *Tie-2* and *survivin* in endothelial cells and down regulated the expression of HIF-1α and VEGF in tumor cells [59]. Yu *et al.* [70], recently described that combination of the multiple receptor tyrosine kinase inhibitor AEE788 with numerous HDACI (panobinostat, LAQ824 and TSA). These combinational strategies resulted in synergistic cytotoxicity in numerous solid and hematological cancer cell lines. AEE788 inhibition of mitogen activated protein kinase (MAPK) and Akt signaling enhanced HDACI-mediated apoptosis via the induction of ROS [70]. 

Tyrosine kinase signaling often results in the activation of survival pathways mediated by PI3K/Akt/mTOR signaling and for this reason has made this pathway attractive for therapy intervention. By using various techniques like pharmacological inhibition using LY294002 (targeting PI3K) and rapamycin (targeting mTOR) as well as biochemical methods including dominant negative expression of Akt, PI3K and PTEN it was observed that the induction of VEGF and HIF-1α was inhibited, linking the PI3K/Akt/mTOR pathway , HIF-1α and angiogenesis [71]. Only recently though has the combination of mTOR inhibition with HDACI been evaluated [72]. Utilizing the capabilities of rapamycin and panobinostat to inhibit HIF-1α through different mechanisms, combination treatment demonstrated greater decrease in clonogenic survival as well as significantly lowering HIF-1α protein expression compared to single agents in PC3, C2 and HUVEC cell lines. In addition the combination of these agents resulted in significant inhibition of PC3 and C2 *in vivo* tumor growth and angiogenesis assessed by tumor weights and microvessel density [72]. 

The concomitant treatment using HDACI with demethylating agents has shown greater anti-tumor effect linked to the inhibition of angiogenesis. Maspin, a member of the serpin superfamily whose activity regulates such biological pathways including angiogenesis and metastasis was shown to have its expression silenced in oral cancer cell lines [73]. Upon treatment with the demethylating agent 5-aza-dC and/or the HDACI FR901228 the re-expression of maspin mRNA was observed [73]. Of interest the re-expression of maspin was not a result of the demethylation of CpG islands, indicating that histone post-translational modifications maybe the key mechanism behind maspin expression. Hellebrekers *et al.* [74] experiments also demonstrated that HDACI and demethylating agents could inhibit immune escape of tumor conditioned endothelial cells *in vitro* and *in vivo* by the re-expression of intercellular adhesion molecule-1 (ICAM-1) restoring leukocyte-endothelial cell adhesion. Hellebrekers *et al.* [69] further discussed the silencing of novel genes which may mediate neo-angiogenesis in tumor conditioned endothelial cells, one of which was clusterin. An additional study carried out by Suuronen *et al.* consolidates that clusterin mediates neo-angiogenesis. Treatment of the human cell line, retinal pigment epithelial cells (ARPE-19) with HDACI valproic acid or TSA and the demethylating agent 5-aza-2’-deoxycytidine resulted in increased clusterin mRNA and protein levels [75], further demonstrating that clusterin expression is epigentically regulated and may play a vital role in neo-angiogenesis in a tumor setting.

**Table 2:** Pro- and anti-angiogenic genes altered by HDACI in cancer and endothelial cells.

Gene	Target Cell	Activity on angiogenesis	Effect on gene transcription by HDAC inhibition [reference]
p53	Cancer	Inhibits	Up-regulation [49]
pVHL	Cancer	Inhibits	Up-regulation [49,58]
HIF-1α	Cancer	Induces	Down-regulation [49,59,60]
VEGF	Cancer	Induces	Down-regulation [49,59,60,61]
Activin A	Cancer	Inhibits	Up-regulation [57]
bFGF	Cancer	Induces	Down-regulation [60,61]
Thrombospondin 1	Cancer	Inhibits	Up-regulation [62,63]
MMP-2	Cancer	Induces	Up-regulation [57]
MMP-9	Cancer	Induces	Up-regulation [57]
RECK	Cancer	Inhibits	Up-regulation [57]
Neurofibromin2	Cancer	Inhibits	Up-regulation [58,64]
Ang1	Cancer	Induces	Down-regulation [38]
Connective tissue growth factor	Cancer	Inhibits	Up-regulated [63]
Fibroblast growth factor 19	Cancer	Induces	Down-regulated [63]
VEGF receptor 1	Endothelial	Induces	Down-regulation [57]
VEGF receptor 2	Endothelial	Induces	Down-regulation [57]
Neuropilin-1	Endothelial	Induces	Down-regulation [57]
Semaphoring III	Endothelial	Inhibits	Up-regulation [65]
Tie2	Endothelial	Induces	Down-regulation [59]
Ang2	Endothelial	Induces	Down-regulation [59]
eNOS	Endothelial	Induces	Down-regulation [66,67,68]
VEGFD	Endothelial	Induces	Down-regulation [57]
Clusterin	Endothelial	Inhibits	Up-regulation [69]
Fibrillin1	Endothelial	Inhibits	Up-regulation [69]
Quiescin Q6	Endothelial	Inhibits	Up-regulation [69]
PDGF-B	Endothelial	Inhibits	Up-regulation [56]
PDGFR-β	Endothelial	Inhibits	Up-regulation [56]
Survivin	Endothelial	Induces	Down-regulation [59]

## 5. HDACIs and Autophagy

Autophagy is an evolutionarily conserved process in which cellular proteins and organelles are sequestered in autophagosomes and ultimately degraded following their fusion with lysosomes. This enables cells to recoup ATP and other molecules critical for biosynthetic pathways during nutrient deprivation or exposure to hypoxia [76]. Recent work as demonstrated that autophagy can be regulated in a p53 induction of DRAM (damage-regulated autophagy modulator) dependent mechanism [77] and also independent of DRAM via execution of p73 signaling [78]. Further, autophagy regulation is also mediated through the inhibitory actions of mTOR on p73 transcriptional activity [79,80]. To date, the role of autophagy in cancer and its potential as a therapeutic target is still a controversial one. Currently the literature discussing autophagy still demonstrates that it can play a role in resistance and/or sensitivity to chemotherapy agents including HDACI. 

Studies to date have identified HDAC1 and HDAC6 to have direct involvement in autophagy [81,82]. Interestingly, only inhibition of HDAC1 resulted in induction of autophagy, whereas HDAC6 was shown to regulate the formation of the autophagic machinery to destroy protein aggregates [44,81]. Induction of HDACI-mediated autophagy was first reported by Shao *et al.* where they observed that either sodium butyrate or vorinostat could induce apoptosis and autophagy in HeLa cells. Inhibition of HDACI-mediated apoptosis occurred by the over-expression of Bcl-X_L_, though this did not diminish HDACI autophagy inducing capabilities [83]. More recently, it was discussed that autophagy may induce resistance to vorinostat in CML cell lines. Treatment of CML cell lines with vorinostat resulted in autophagy which diminished vorinostat-mediated apoptosis. When these cell lines were co-treated with the autophagy inhibitor chloroquine and vorinostat, vorinostat induced apoptosis was restored [84]. Later, Carew and colleagues demonstrated in a colon cancer model that the combination strategy of chloroquine and vorinostat resulted in increased vorinostat mediated apoptosis. Of interest knockdown of HDAC6 only marginally sensitized cells to chloriquine [76]. Importantly, it was also demonstrated that this combinational approach reduced tumor burden and increased apoptosis in a colon cancer xenograft model [76]. Another study by Walker *et al.* also noted that concurrent treatment of colon cancer cell lines with sorafenib and vorinostat activated the c-Jun NH(2)-terminal kinase pathway which disassociated Beclin-1 from Bcl-2, promoting autophagy. Knockdown of Beclin-1 inhibited autophagy and increased chemotherapy mediated toxicity [85]. Interestingly, treatment of HeLa S3 cells with vorinostat resulted in autophagic cell death. This cell death was characterized by the inhibition of mTOR activity and the up-regulation of two documented autophagy genes, *BECLIN-1* and *ATG-7* [86]. In addition, Hrzenjak *et al.* also observed that vorinostat reduced mTOR expression and induced caspase-independent toxicity indicative of autophagy in endometrial stroma sarcoma cells [87]. Finally, Ellis and colleagues [88] demonstrated that LAQ824 or panobinostat treatment of Eμ-myc lymphomas *in vitro* and *in vivo* devoid of a functional apoptosome, either through deletion of *apaf-1* or *caspase-9* did not result in resistance to these agents and failed to inhibit their therapeutic activities. Further analysis revealed that Eμ-myc lymphomas devoid of a functional apoptosome displayed morphologic and biochemical features of autophagy with LAQ824 or panobinostat, and most importantly indicating that HDACI-mediated autophagy results in a therapeutic response (Table 3). 

**Table 3:** Apoptotic and Autophagy related genes regulated by HDACI.

Biological effect/gene	Pathway	Effect on gene transcription by HDAC inhibition [reference]
**Autophagy**		
Beclin-1	Aggresome	Up-regulated [86]
ATG-7	Aggresome	Up-regulated [86]
**ROS production/activity**		
TBP2	ROS	Up-regulated [104]
Thioredoxin	ROS	Up-regulated [104]
**Apoptosis**		
TRAIL	Extrinsic apoptosis	Up-regulated [105,106,107,108]
DR5	Extrinsic apoptosis	Up-regulated [105,106,107,108]
DR4	Extrinsic apoptosis	Up-regulated [109]
Fas	Extrinsic apoptosis	Up-regulated [106,110]
FasL	Extrinsic apoptosis	Up-regulated [106,110]
TNFα	Extrinsic apoptosis	Up-regulated [111]
c-FLIP	Extrinsic apoptosis	Down-regulated [101,103]
Bcl2	Intrinsic apoptosis	Down-regulated [112]
BclXL	Intrinsic apoptosis	Down-regulated [31,113]
Bclw	Intrinsic apoptosis	Down-regulated [114]
Mcl-1	Intrinsic apoptosis	Down-regulated [31,113]
XIAP	Intrinsic apoptosis	Down-regulated [115,116]
Caspase-3	Intrinsic apoptosis	Up-regulated [31]
Apaf-1	Intrinsic apoptosis	Up-regulated 31]
Bak	Intrinsic apoptosis	Up-regulated [31,104,113]
Bid	Intrinsic apoptosis	Up-regulated/cleaved [116,117,118]
Bim	Intrinsic apoptosis	Up-regulated/phosphorylated [104,113,118,119,120]
Bmf	Intrinsic apoptosis	Up-regulated [104,121]
Bax	Intrinsic apoptosis	Up-regulated/phosphorylated [119]
Noxa	Intrinsic apoptosis	Up-regulated [120]
Puma	Intrinsic apoptosis	Up-regulated [122]
AVEN	Intrinsic apoptosis	Down-regulated [63]
Survivin	Intrinsic/Extrinsic apoptosis	Down-regulated [123]

## 6. HDACIs and Apoptosis

Apoptosis, also known as programmed cell death, is a regulated process important in tissue homeostasis and development. Apoptosis is morphologically characterized by plasma membrane blebbing, cell shrinkage, chromatin condensation, phosphatidylserine exposure, DNA degradation and fractionation of the cell into smaller vesicles which are engulfed by phagocytes [89,90]. The apoptotic pathways are tightly regulated at a number of levels and dysregulation of apoptosis can result in the manifestations of human disease, including cancer [91,92]. Apoptosis is executed by the activation of cysteine proteases known as caspases [93] mediated by two functionally distinct, yet molecularly linked, apoptotic pathways, the death receptor (extrinsic) pathway and the mitochondrial (intrinsic) pathway [94]. HDACI induce tumor cell death with all the biochemical and morphological characteristics of apoptosis, and it appears that that HDACI-induced apoptosis involves transcription-dependent and transcription-independent mechanisms [5,95,96,97,98]. Over the years multiple papers have been published identifying genes both regulated and important to HDACI mediated apoptosis. For a summary of apoptotic genes regulated by HDACI refer to Table 3 and references within. 

### 6.1. Combination Strategies with HDACI to Target Apoptosis

Altered levels of anti-apoptotic proteins in cancer cells have demonstrated to drive resistance against HDACI-mediated apoptosis. More specifically it was observed that by inhibiting the transcriptional activity of the JAK/STAT pathway in CTCL patients, one could re-sensitize resistant CTCL cells to vorinostat induced apoptosis [99]. This data possibly indicates the importance of HDACI-mediated apoptosis correlation to therapeutic efficacy.

Because HDACI have the ability to alter the expression of apoptotic proteins combination strategies are being tested in effort to increase apoptosis within tumor cells. In the case of the death receptor signaling pathway HDACI in combination with TRAIL (tumor necrosis factor-related apoptosis inducing ligand) or anti-TRAIL receptor agonists seem most promising due to both agents being tumor-selective and relatively non-toxic to non-malignant cells [100]. *In vitro* low dose concentrations of HDACI have been recently shown to sensitize malignant cells to TRAIL-induced apoptosis [101], and more importantly this combination strategy was observed to be ineffective in inducing TRAIL-mediated apoptosis in the treatment of non-malignant cells [102]. Frew and colleagues also went onto to demonstrate that concurrent treatment of a syngeneic mouse model of breast cancer with vorinostat and MD5-1 (agonistic anti-TRAIL receptor antibody) resulted in significant therapeutic responses *in vivo* [101]. Excitingly, only combination treated mice achieved complete regression of tumor growth. This synergistic therapeutic efficacy was achieved by the induction of apoptosis secondary to proteosome-mediated down regulation of c-FLIP, with no change in TRAIL or death receptor expression observed [101]. Moreover, Panobinostat treatment sensitized TRAIL resistant pancreatic cancer cell lines to TRAIL mediated apoptosis. Further, it was concluded that TRAIL induced apoptosis was mediated by panobinostat’s ability to increase levels of ubiquitinated c-FLIP and its proteasomal degradation [103]. 

An alternate combination strategy investigating the augmentation of apoptosis is the use of HDACI with inhibitors of the pro-survival Bcl-2 proteins, which are commonly over-expressed in cancer and mediate resistance to apoptosis induction by chemotherapy agents [124]. *In vivo* therapeutic efficacy was not achieved in mouse models treated with vorinostat, LAQ824 or panobinostat, due to the inhibition of HDACI-mediated apoptosis through the over-expression of Bcl-2 [88,118]. This resistance to HDACI-mediated apoptosis was recently observed to be overcome by the combinational treatment of vorinostat and ABT-737 (the small molecule inhibitor of Bcl-2) *in vitro* and *in vivo* [125]. An ABT-737 homolog, ABT-263 has been developed and is orally bioavailable and is currently under investigation in clinical trials [126,127]. This data demonstrates the great potential for this rational combination strategy in human patients that may have developed refractory disease due to the up-regulation of anti-apoptotic Bcl-2 proteins (Figure 1). 

A recent report from Mahalingam and colleagues demonstrated that treatment of two renal cell carcinoma xenograft murine models with vorinostat and temsirolmus resulted in greater anti-tumor activity when these agents were administered concurrently [123]. Further, while each agent alone lead to a modest decrease in the pro-angiogenic/anti-apoptotic protein survivin, it was the combinational therapy that dramatically reduced its expression. The loss of survivin resulted in the induction of apoptosis and also a strong reduction in angiogenesis [123]. An interesting point that was not covered by the authors in this paper was the relationship between inhibition of angiogenesis and induction of apoptosis, and whether these responses are dependent or independent from each other. 

## 7. Clinical Combination Strategies including HDACI

To date multiple HDACI have advanced to clinical development and have been entered in clinical trials. As already mentioned, vorinostat and romidepsin are the first HDACI to be approved by the FDA as clinical therapies for patients with CTCL. Further, multiple clinical trails are currently underway investigating the clinical potential of HDACI as monotherapies in numerous cancers [128]. A major focus for researchers and clinicians alike is to advance the clinical use of HDACI in rationale combination strategies.

**Figure 2:**
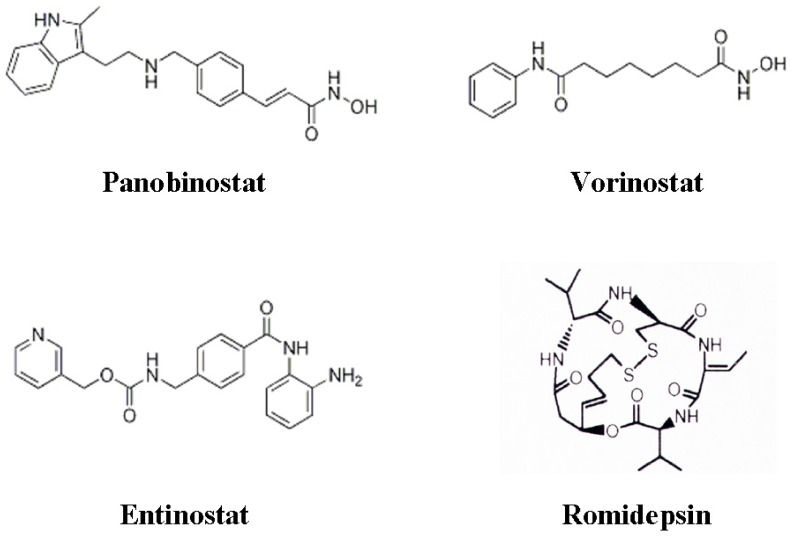
Chemical structures of clinically relevant HDAC inhibitors.

Numerous phase I studies involving vorinostat (Figure 2) in combination with established chemotherapy agents have recently been reported in advanced or refractory solid tumors and haematological malignancies [129]. One such study includes the combination of vorinostat with carboplatin and paclitaxel. Twenty five from twenty eight (25/28) patients with advanced solid tumors were available for evaluation. Of interest was the promising activity of this combination strategy in patients with advanced NSCLC, with 10/19 (53%) achieving a partial response and 4/19 (21%) achieving stable disease [130]. Another phase I study was recently reported including patients with refractory colorectal cancer who were treated with vorinostat in combination with FOLFOX (5-fluorouracil, leucovorin and oxalplatin). This study was conducted to determine the maximum tolerated dose (MTD) of vorinostat in combination with fixed doses of FOLFOX. At the studies conclusion it was observed the MTD of vorinostat in this combinational strategy was 300 mg orally twice daily (×1 week for 2 weeks) [131]. Munster and colleagues investigated the clinical efficacy of vorinostat in combination with the topoisomerase inhibitor doxorubicin in patients with solid tumors [132]. In this phase I study a total of 32 patients were treated, 24 of which were available for evaluation. This combination strategy produced a best clinical outcome of two partial responses in patients with breast and prostate cancer and two stable disease responses in melanoma patients. Correlative studies confirmed that changes in histone hyperacetylation were comparable between peripheral blood mononuclear and tumor cells. Of interest, Munster *et al.* noted that HDAC2 maybe useful both as both as a response and prediction biomarker. Expression of HDAC2 correlated with prediction to histone hyperacetylation. This observation raises the possibility of specifically targeting HDAC2 with iso-specific inhibitors [132]. Another phase I study investigated the combinational potential of vorinostat with docetaxel in patients with castrate resistant prostate cancer (CRPC), relapsed urothelial or non-small cell lung carcinoma (NSCLC). Unfortunately, this study was terminated due to poor toleration of drug schedule and excessive dose limiting toxicities (DLTs) by patients. DLTs included grade 4 neutropenic fever/sepsis, myocardial infarction and GI bleed [133]. Vorinostat in combination with gemcitibine and cisplatinum results were recently reported from a phase I trail in NSCLC patients to investigate the DLTs and maximum tolerated dose (MTD). Of the 28 patients currently enrolled, 19 patients were available for evaluation, of which nine (47%) reached a partial response. It was concluded that vorinostat could be administered safely with standard doses of gemicitibine and cisplatinum in patients with metastatic NSCLC [134]. A further study has recently trialed vorinostat treatment in patients that had developed resistance to the tyrosine kinase inhibitor erlotinib by development of EGFR mutations. At present four patients have discontinued combination therapy due to disease progression, but of the nine patients currently available for evaluation, six (67%) patients had stable disease as best response, indicating that combination of vorinostat and erlotinib overall is well tolerated and clinically effective. These promising results give hope to NSCLC patient’s refractory to erlotinib due to EGFR mutations and can be re-sensitized with the addition of vorinostat [135]. More recently, reports have emerged discussing findings from two phase I trials involving vorinostat in combination treatment strategies of patients with solid tumors. A dose escalation study involving vorinostat in combination with sorafenib was carried out to determine the MTD and recommended phase II dose (RP2D). At the studies conclusion 12/17 patients representing various solid malignancies were available for evaluation. It was concluded that the MTD/RP2D combination dose of 300 mg Vorinostat (QD, days 1–14) and 400 mg Sorafenib (BID, days 1–21) in 21 day cycles. No drug related deaths were reported and one patient (renal sarcoma) reached an unconfirmed PR, while nine patients reached SD with minor responses (Table 4) [145]. The approved oral dose of vorinostat (400 mg/day) is inconsistent at enhancing chemotherapy. In effort to safely increase vorinostat serum levels (>2.5 μM) a phase I study was initialized to investigate the intermittent oral pulse-dose in combination with flavopiridol in patients with advanced solid tumors. Of the recruited 34 patients, 31 were available for response evaluation. With a best response of stable disease (eight patients), this combination schedule concluded that intermittent high dose of vorinostat in combination with flavopiridol was feasible and achieved vorinostat serum levels >2.5 μM with no increase in already documented toxicities. It was determined that the RP2D dose is 800 mg vorinostat (once daily for 3 days [days 1–3]) with 30 mg/m^2^ flavopiridol (over 30 minutes followed by 30 mg/m^2^ over 4 hours, every 14 days) [146]. 

**Table 4:** Summary of current clinical trails involving combination strategies with HDACI.

HDACI	Combination	Phase	Disease	Patient number	Response
**Vorinostat**	Carboplatin/Paclitaxel	I	Advanced solid tumors	25/28 patients available for evaluation	NSCLC patients were best responders; PR (53%), SD (21%)
	FOLFOX	I	Refractory colorectal cancer	21 patients enrolled	Study resulting in a determined vorinostat MTD of 300 mg 2× daily in combination with FOLFOX
	Doxorubicin	I	Solid tumors	24/32 patients available for evaluation	PR (8%; prostate and breast cancer patients); SD (8%; melanoma patients)
	Docetaxel	I	CRPC and NSCLC	NA	Study terminated due to excessive DLTs
	Gemcitibine/cisplatinum	I	Metastatic NSCLC	19/28 patients available for evaluation	PR (47%)
	Erlotinib	I	Refractory NSCLC	9 patients available for evaluation	SD (67%)
	Bortezomib	I	Refractory solid tumors	29 patients available for evaluation	Study resulted in a determined vorinostat MTD of 300 mg BID with bortzomid dosed at 1.3 mg/m^2^. Evidence of clinical activity was observed
	Bevacizumab	II	Stage IV clear cell renal carcinoma	32/34 patients available for evaluation	18% objective responses (1× CR; 5× PR), 67% (SD). Median progression free survival: 5.3 months Overall survival: 16.2 months
	Sorafenib	I	Advanced solid tumors	12/17 patients available for evaluation	1 unconfirmed PR; 9 SD (minor responses). MTD/RP2D in combination recommended is 300 mg vorinostat QD d 1–14 with 400 mg sorafenib BID d 1–21 (21 day cycles).
	Flavopiridol	I	Advanced solid tumors	31/34 patients evaluable for evaluation	Concluded that intermittent pulsing of high dose vorinostat in combination with flavopiridol is achievable without increased toxcities. RP2D is 800 mg vorinostat (3 days; d 1–3) with 30 mg/m^2 ^flavopiridol (30min followed by 30 mg/m^2^ every over 4h every 14d).
**Romidepsin**	Gemcitibine	I	Advanced solid tumors	33 patients available for evaluation	SD (36%)
	Bortezamib	II	Refractory/relapsed multiple myeloma	5 patients currently enrolled	Concluded that this combination is active and further patient recruitment is currently underway
**Entinostat**	Erlotinib	I	Advanced NSCLC	9 patients available for evaluation	PR (11%) and SD (11%)
	5-azacitidine	II	Relapsed advanced NSCLC	25 patients currently enrolled	CR (4%) and SD (8%); remaining patients had PD
	Aromatase inhibitor therapy	II	ER+ breast cancer	27 patients enrolled	1 confirmed PR; 1 SD > 6 months. Concluded this combination demonstrated clinical benefit.
**Panobinostat**	Trastuzumab	I	HER2 positive metastatic breast cancer	18 patients enrolled	Preliminary data indicates this combination to be well tolerated and displays clinical activity
	Lenalidomide/ dexamethasome	I	Relapsed/refractory multiple myeloma	22 patients enrolled	Combination well tolerated with indications of clinical efficacy
	Docetaxel	Ib	Chemotherapy naïve CRPC	21 patients enrolled	Minimal DLTs have been observed with some patients achieving a biochemical response indicated by reduced PSA levels
	Epirubicin	I	Solid tumors	10 patients	Patient cohort treated with 50 mg panobinostat reported to date and concluded that sequence combination of panobinostat and epirubicin is well tolerated.

Within our laboratory, a recent clinical trial investigating the combination of vorinostat with bevacizumab was reported. Patients with stage IV clear cell renal carcinoma (RCC) previously treated with VEGF receptor tyrosine kinase inhibitors were recruited. Treatment consisted of vorinostat at 200 mg orally twice daily over 2 weeks and bevacizumab at 15 mg/kg intravenously every 3 weeks. To date 32/34 patients were evaluable for evaluation. Within the completion of this phase II study, two patients experienced DLTs consisting of grade 4 thrombocytopenia and three patients with grade three thromboembolic events. Excitingly six objective responses (18%) were observed including one complete response and five partial responses. Nineteen (67%) patients have stable disease, with a median progression free survival and overall survival of 5.3 months and 16.2 months respectively. Correlative studies demonstrated a possible association with lower HIF-1α staining and objective responses. It was concluded that the combination of vorinostat (200 mg PO BID) with bevacizumab (15 mg/kg) was well tolerated and produced objective clinical responses in patients with RCC [144] (Table 4; Figure 1). Finally, patients with refractory solid tumors have been entered into a phase I trail to investigate the potential of the combination approach of bortezomib and vorinostat. Although grade 3/4 DLTs were reported which included thrombocytopenia (17%) and diarrhea (10%), it was concluded that combination of bortezomib and vorinostat resulted in subjective clinical activity in patients with refractory solid tumors [136]. 

While romidepsin (Figure 2) has been approved by the FDA to treat patients with CTCL, combination strategies have also been recently reported. The combination of romidepsin and gemcitibine was recently evaluated in patients with advanced solid tumors. This report consisted of 33 patients which have received over 104 cycles of the combination regime, made up of a 4 hour infusion of romidepsin followed by gemcitibine over 30 minutes on days 1, 8 and 15 of a 28 day cycle. Reported toxicities have been mild to moderate with a best clinical response being stable disease observed in 12/33 (36%) patients after ≥4 cycles [137]. A further phase II study of a 1 hour infusion of romidepsin combined with bortezomib in multiple myeloma (MM) patients with refractory or relapsed disease was conducted. Currently a total of 5 patients have been enrolled with two patients achieving a minimal response after two cycles. Although two patients have experienced a grade 3 DLT (thrombocytopenia), it was concluded that this combinational schedule is active for MM patients with refractory or relapsed disease, and therefore further recruitment of patients is still ongoing [138].

Another class I HDAC selective inhibitor, entinostat (Figure 2) which is structurally different to romidepsin has been recently reported in combination therapeutic strategies. A recent phase I study investigating the combination of erlotinib and entinostat in the treatment of patients with advanced NSCLC was reported. Patients received either a dose of 5 or 10 mg of entinostat once every two weeks with 150 mg erlotinib daily. From these dosing schedules the most common adverse events were anorexia and asthenia. Of the enrolled nine patients for evaluation, there was one confirmed partial response and another one with stable disease. The safety profile from this study has allowed for the progression of a phase II study in patients with advanced NSCLC [139]. Another promising combination strategy with HDACI is the addition of DNA methyltransferase inhibitors (DNMTI) such as 5-azacitidine (5AC). A phase II study of entinostat combined with 5AC was recently investigated and reported in patients with relapsed advanced NSCLC. Patients received a fixed 7 mg/day dose of entinostat which was given on days 3 and 10 and 5AC was administered on days 1–6 and 8–10 at a dose of 40 mg/m^2^. Currently, 25 patients have been currently enrolled in this study and one patient had a complete response and is currently disease free at 20 months with two further patients reaching stable disease; the remaining patients had progression of disease. This clinical trial showed that this combination strategy is well tolerated and safe with evidence of clinical activity in patients with advanced NSCLC [140]. 

A phase II study recently investigated the addition of entinostat in women with estrogen receptor positive breast cancer that were progressing while being treated with aromatase inhibitors (AI). Patients continuing with their AI therapy received an additional entinostat dose of 5 mg/week in a 28 day cycle. Common low grade toxicities included were nausea, diarrhea and fatigue with grade ≥3 toxicities being diarrhea, dyspenia, fatigue and lethargy, though no DLTs where reported. Interestingly, increased protein acetylation was reported in immune cells including CD8+, CD14+ and CD19/20+ cells. An increase in peripheral mononuclear cell apoptosis was also noted but the specific anti-tumor activity and drug effect on these cells was not elaborated on. Of the 27 patients enrolled, one confirmed PR and one SD greater than 6 months was reported and concluded that is combination treatment in patients with progressive breast cancer demonstrated clinical benefit [148]. 

Panobinostat (Figure 2) has shown great promise as a monotherapy in CTCL [38], but combinational strategies from clinical trials are starting to be reported with exciting preliminary results. A phase I trial of patients with HER2 positive metastatic breast cancer was pretreated with either IV administration (Day 1 + 8) or oral panobinostat (three times a week, continuously) every 3 weeks with a standard dose of trastuzumab weekly. Of the two panobinostat regimes only one DLT was reported, which was a grade 4 thrombocytopenia in the oral arm. To date preliminary results have demonstrated that this combination is well tolerated and displays promising clinical activity [141]. A further phase I trial, this time investigating panobinostat in combination with lenalidomide and dexamethasone in patients with multiple myeloma (MM) has been conducted. Twenty two patients with relapsed or relapsed refractory MM were treated with fixed doses of lenalidomide and dexamethasone combined with dose escalation of panobinostat. Currently the 5 and 10 mg dose of panobinostat in this triple oral combination appear safe with indications of clinical efficacy [142]. Panobinostat has also been recently investigated in combination with docetaxel in a phase Ib study. Twenty one chemotherapy naïve patients with castration resistant prostate cancer (CRPC) were treated with escalating doses of panobinostat (10, 15 and 20 mg/m^2^) administered IV on days 1 and 8 with a fixed dose of docetaxel (75 mg/m^2^) on day 1 and prednisone (5 mg) in a 21 day cycle. While no conclusive clinical results are currently available it appears that combination of panobinostat at 10 mg and 15 mg/m^2^ with docetaxel is achievable in patients with minimal DLTs. A promising observation was that some patients achieved a reduction in PSA levels from baseline [143]. Another phase I trial investigated the ability of panobinostat to potentiate epirubicin induced apoptosis in patients with solid tumors. This involved escalating doses of oral panobinostat (20–50 mg, days 1, 3 and 5 for a total of 3 weeks), followed by IV administration of epirubicin at 75 mg/m^2^ at day 5. Current evaluation of the panobinostat 50 mg cohort is available with DLT toxicities including grade 3 atrial fibrillation with rapid ventricular response, while non-limiting grade 3 and 4 toxicities included neutropenia, thrombocytopenia and fatigue. Stable disease was achieved in one patient each with melanoma, ovarian and neuroblastoma of the nine patients evaluated [147].

The clinical development of HDACI poses some relevant questions to investigators. The dose and schedule of these agents still needs to be optimized. The correct dosing represents a major challenge in particularly for the combination strategies. If the biological endpoint is to induce tumor cell apoptosis therapeutic strategies with higher doses of HDACI in a pulsed fashion should be pursued. In contrast, if gene/protein modulation is the goal, perhaps low doses HDACI but chronically administered might be clinically effective. 

## 8. Concluding Remarks

Over the past decade a better understanding of epigenetic mechanisms that attribute to tumorigenesis has created a great opportunity for the clinical development of novel epigenetic targeted therapies. More so, altered HDAC activity and their mediated changes in the epigenetic programming that possibly drives the emergence of refractory disease has become a leading target for drug discovery/development through their inhibition. The use of HDACI as single agent treatments in both pre-clinical tumor models and clinical trials has returned promising results to indicate these compounds are well tolerated and clinically active. While some clinical studies have shown long term administration associated with clinical response of HDACI as monotherapy, unfortunately resistant and/or the reversal of drug efficacy (often from treatment withdrawal), is constantly observed. Excitingly, HDACI have demonstrated the ability to augment the cytotoxic effects of other targeted therapies or re-sensitize a tumor to a therapy it has become refractory towards. Because of these promising capabilities of HDACI in combinational strategies discussed above, it is believed that this is where HDACI will be best utilized in a clinical setting. One question that still remains unanswered, and is currently being investigated by our laboratory and others work, is whether concurrent or sequential combination with other treatments is most beneficial to the patient. This answer may be tumor and/or stage specific, but is the belief of the writers that HDACI in combination therapies will maximize clinical efficacy of this novel class of agents in the treatment of cancer patients. 
